# Proteomic features of soft tissue tumours in adolescents and young adults

**DOI:** 10.1038/s43856-024-00522-x

**Published:** 2024-05-18

**Authors:** Yuen Bun Tam, Kaan Low, Hari PS, Madhumeeta Chadha, Jessica Burns, Christopher P. Wilding, Amani Arthur, Tom W. Chen, Khin Thway, Anguraj Sadanandam, Robin L. Jones, Paul H. Huang

**Affiliations:** 1 Division of Molecular Pathology, The Institute of Cancer Research, London, United Kingdom; 2 Department of Oncology, National Taiwan University Hospital, Taipei, Taiwan; 3 Graduate Institute of Oncology, National Taiwan University College of Medicine, Taipei, Taiwan; 4 The Royal Marsden NHS Foundation Trust, London, United Kingdom; 5 Division of Clinical Studies, The Institute of Cancer Research, London, United Kingdom

**Keywords:** Sarcoma, Paediatric cancer

## Abstract

**Background:**

Adolescents and young adult (AYA) patients with soft tissue tumours including sarcomas are an underserved group with disparities in treatment outcomes.

**Methods:**

To define the molecular features between AYA and older adult (OA) patients, we analysed the proteomic profiles of a large cohort of soft tissue tumours across 10 histological subtypes (AYA n = 66, OA n = 243), and also analysed publicly available functional genomic data from soft tissue tumour cell lines (AYA n = 5, OA n = 8).

**Results:**

Biological hallmarks analysis demonstrates that OA tumours are significantly enriched in MYC targets compared to AYA tumours. By comparing the patient-level proteomic data with functional genomic profiles from sarcoma cell lines, we show that the mRNA splicing pathway is an intrinsic vulnerability in cell lines from OA patients and that components of the spliceosome complex are independent prognostic factors for metastasis free survival in AYA patients.

**Conclusions:**

Our study highlights the importance of performing age-specific molecular profiling studies to identify risk stratification tools and targeted agents tailored for the clinical management of AYA patients.

## Plain language summary

Soft tissue tumours are cancers that develop in the connective and supporting tissues of the body, such as muscle or fat. These tumours arise in patients across the entire age range. However, improvements in survival outcomes in adolescent and young adult (AYA) patients have lagged behind outcomes in older adults (OA) and children. To better understand the biology of AYA patients with soft tissue tumours, we analysed protein profiles across 10 different types. We identified biological differences between AYA and OA patients and report an age-specific signature that can potentially be used to help predict which AYA patients are more likely to have aggressive cancers that will spread to other parts of the body. Our study highlights the importance of performing age-specific studies to identify new tools to predict patient outcomes and potentially find more suitable treatments.

## Abstract (web summary)

Tam et al. compare molecular features between adolescents and young adult (AYA) and older adult (OA) patients with soft tissue tumours and identify that components of the spliceosome complex are independent prognostic factors for metastasis free survival in AYA patients.

## Introduction

Soft tissue tumours are rare mesenchymal tumours that span >80 histological subtypes of distinct biology and genetics^1^. These include malignant cancers such as sarcomas as well as tumours with no metastatic potential such as desmoid tumours (DES). Sarcomas have a higher incidence in cancers diagnosed in the adolescent and young adult (AYA) age group (16–39 years at the time of diagnosis, 8% of all cancer diagnosis), compared to older adults (OA) (>39 years, 1% of all cancer diagnosis)^2,3^. Despite the increased incidence, improvements in survival in AYA patients with soft tissue sarcomas (STS) have lagged behind other age groups^4^. Reasons for this disparity are multi-factorial, and include under-representation in clinical trials^4,5^, unique psychosocial considerations^6,7^, inadequate age-specific services^8–10^, and poor knowledge of their unique biology^11,12^. A recent study showed that STS histological subtypes typically sensitive to chemotherapy in other age groups are instead chemoresistant in AYA patients^13^, which suggests that patients in this age group may have distinct biological differences compared to either OA or paediatric patients. In the case of non-rhabdomyosarcoma STS (NRSTS) where the majority of current treatment guidelines relies on drugs that have been optimised in OA patients^14,15^, the lack of therapies tailored to AYA patients is a major unmet need and a key barrier to improving survival rates.

Several recent pan-cancer analyses have leveraged publicly available datasets (TCGA, ICGC, GENIE) to demonstrate that there are age-associated genomic, transcriptomic and immune microenvironmental differences across multiple cancer types that include STS^16–20^. For instance, Lee et al., showed that sarcoma patients <50 years old had lower immune-related pathway expression compared to patients >50 years of age at the transcriptomic level^17^. They further determined that at the genomic level, the older sarcoma patients had higher copy number variation rates. However, the AYA age group is under-represented in all these studies, with only a small number of STS patients and histological subtypes included. Furthermore, these aggregate analyses do not consider well-established differences in the spectrum of STS histological subtypes in AYA versus OA patients^21^. Prior studies that have undertaken molecular profiling of AYA sarcoma specimens including a recent EORTC SPECTA-AYA study have focused exclusively on genomic and transcriptomic data^20,22,23^. While informative, these technologies do not provide a direct measure of proteins which are key mediators of tumour cell signalling and the largest class of targets for oncology drugs^24–27^, making it challenging to bridge the translational gap towards clinical applications. Given the unique tumour, microenvironmental and host differences between AYA and OA patients^12^, it is likely that AYA patients with STS harbour distinct molecular features which may influence clinical and treatment outcomes, although this has yet to be conclusively demonstrated. Due to the rarity and heterogeneity of STS, to date, there are few studies that have systematically evaluated the molecular differences between AYA and OA patients.

Here, we undertake a detailed analysis of the proteomic features in AYA and OA patients across 10 histological subtypes of soft tissue tumours. By interrogating clinically annotated proteomic profiles in a large cohort of AYA and OA patients and combining it with functional genomics data derived from sarcoma cell lines within the Cancer Cell Line Encyclopaedia (CCLE), we demonstrate that there are significant differences in the biological networks and intrinsic vulnerabilities between these two age groups with implications for biomarker development and therapy selection.

## Methods

### Patient cohort

The cohort is comprised of 309 patients from two centres (The Royal Marsden Hospital and National Taiwan University Hospital). Patients with a histopathologically confirmed diagnosis of soft tissue sarcoma (STS) or desmoid tumour, and 16 years of age or older at the time of diagnosis were included in the analysis. Soft tissue sarcoma diagnoses included angiosarcoma, alveolar soft part sarcoma, clear cell sarcoma, dedifferentiated liposarcoma, desmoplastic small round cell tumour, epithelioid sarcoma, synovial sarcoma, leiomyosarcoma, and undifferentiated pleomorphic sarcoma. Retrospective collection and analysis of associated clinical data was approved as part of the Royal Marsden Hospital (RMH) PROgnoStic and PrEdiCTive ImmUnoprofiling of Sarcomas (PROSPECTUS) study (NHS Research Ethics Committee Reference 16/EE/0213) or National Taiwan University Hospital (Research Ethics Committee Reference 201912226RINB). Written informed consent was obtained from participants, and all participants were old enough to provide informed consent according to local regulations. Baseline clinicopathological characteristics and survival data were collected by retrospective review of medical records as part of our previous study^28^.

### Proteomic data

Proteomic data for this study was downloaded from ProteomeXchange (PXD036226)^28^. The SequestHT search engine in Proteome Discoverer 2.2 or 2.3 (Thermo Scientific, Waltham, MA, USA) was used to search the raw mass spectra against reviewed UniProt human protein entries (v2018_07 or later) for protein identification and quantification. Precursor mass tolerance was set at 20 ppm and fragment ion mass tolerance was 0.02 Da. Spectra were searched for fully tryptic peptides with a maximum of two missed cleavages. TMT6plex at N-terminus/lysine and carbamidomethyl at cysteine were selected as fixed modifications. Dynamic modifications were the oxidation of methionine and deamidation of asparagine/glutamine. Peptide confidence was estimated with the Percolator node. The peptide false discovery rate (FDR) was set at 0.01 and validation was based on *q* value and decoy database search. The reporter ion quantifier node included an integration window tolerance of 15 ppm and an integration method based on the most confident centroid peak at the MS3 level. Only unique peptides were used for quantification, considering protein groups for peptide uniqueness. Peptides with average reporter signal-to-noise >3 were used for protein quantification. Proteins with an FDR <0.01 and a minimum of two peptides were used for downstream analyses.

All data were processed using custom R scripts in R v3.5.1 or later. Proteins identified in <75% of samples were removed, and the remaining missing values were imputed using the k-nearest neighbour (k-NN) algorithm^29^. To normalise the data and remove batch effects, data for each patient sample was divided by the corresponding reference sample and log2 transformed, followed by median centring across samples and standardising within samples. To visualise the STS proteomic dataset, hierarchical clustering was performed using Pearson correlation distance.

### Statistical methods

All statistical tests were two-sided and unless otherwise stated, *p* values were adjusted to false discovery rate (FDR) using the Benjamini–Hochberg (BH) procedure to account for multiple comparisons where required. Unless otherwise specified, analysis was performed using custom R scripts in R v4.1.1 or later. Two-way analysis of variance (ANOVA) and chi-square tests were implemented, with further details of statistical tests listed in the figure legends.

#### Differential expression analysis

To identify upregulated proteins in AYA and OA patients, a two-tailed multiple *t*-test was performed and corrected for multiple comparisons by the BH procedure. Logistic regression analysis was performed to adjust for confounding factors of tumour size, grade, anatomical site, performance status, histological subtype, tumour margin, and tumour depth. Univariate logistic regression first was performed to identify significantly different proteins between AYA and OA (FDR <0.05). Univariate logistic regression was then performed to identify significantly different confounding factors. Each significant protein’s expression was then combined with significant confounding factors and multiple logistic regression was performed with AYA and OA variable.

#### Single sample GSEA (ssGSEA)

Single sample GSEA (ssGSEA) was performed using the GenePattern online tool (www.genepattern.org, v10.1.0) to score sample-specific enrichment of hallmark gene sets (v2023.1), KEGG Spliceosome complex gene set^30^ and Sarcoma Proteomic Module (SPM) gene sets^28^ in the proteomics dataset. ssGSEA score between AYA and OA patients were analysed using a two-way analysis of variance (ANOVA) followed by Šidák correction. Univariate logistic regression was first performed to identify significantly differentially expressed hallmark gene sets between AYA and OA (FDR <0.05). Each significant gene set was then combined with significant confounding factors and multiple logistic regression was performed with AYA and OA variables.

#### Comparative analysis of CCLE functional genomic and proteomics data

Gene Set Enrichment Analysis (GSEA)^30^ was performed using the GenePattern online tool (www.genepattern.org, v20.4.0). To identify MSigDB Reactome gene sets (v2023.1) enriched in the two phenotypic classes (in this case, the OA or AYA age groups) within the functional genomic CCLE dataset, only cell lines with a NRSTS subtype and a recorded patient age of >16 years were included for analysis. Cell lines derived from patients between 16–39 years old were grouped as AYA and above 39 years old as OA. The full list of cell lines included is provided in Supplementary Data 1. Genome-scale CRISPR-Cas9 screening data of cell lines was downloaded from the CCLE portal (https://sites.broadinstitute.org/ccle)^31^. The full list of genes from the CRISPRGeneEffect dataset (DepMap Public 22Q4) was used for GSEA with an FDR <0.05 cut-off for significance. In parallel, GSEA was separately performed on the proteomic dataset to analyse Reactome gene sets that were enriched in soft tissue sarcoma specimens from OA versus AYA patients (FDR <0.05 cut-off). The top enriched gene sets from the proteomic and functional genomic datasets were compared, and shared significant hits were reported.

#### SPM analysis and protein–protein interaction (PPI) networks

PPI networks were built in Cytoscape v3.9.1 or later^32^. Previously described SPMs were utilised to identify proteomic signatures enriched in AYA and OA patients^28^ (Supplementary Data 2). The full SPM network was visualised using protein co-occurrence scores and the group attributes layout using SPM membership. For the full SPM network, a co-occurrence score threshold of >0.05 was applied. To inspect the network of individual spliceosome components, protein networks were constructed using the STRING scores obtained from the STRING database v11.0^33^, with a confidence cut-off score of 0.7 and a grid layout used.

#### Survival analyses

Patients were split into -high or -low expressing groups for SPM6 or spliceosome components based on the median protein expression level. The association of patient groups with survival outcome were evaluated based on Kaplan–Meier survival estimates and univariable Cox analysis with two-sided Wald test. Multivariable Cox analysis was used to adjust for clinicopathological variables. Three survival outcome endpoints were used. Overall survival (OS) is defined as the time from primary disease surgery to death from any cause. Metastasis free survival (MFS) is defined as the time from primary disease surgery to radiologically confirmed metastatic disease or death. Local recurrence free survival (LRFS) is defined as time from primary disease surgery to radiologically confirmed local recurrence or death. Patients who did not have an event were censored at their last follow-up time, up to 60 months.

### Reporting summary

Further information on research design is available in the Nature Portfolio Reporting Summary linked to this article.

## Results

### Cohort and clinicopathological data

**Fig. 1:**
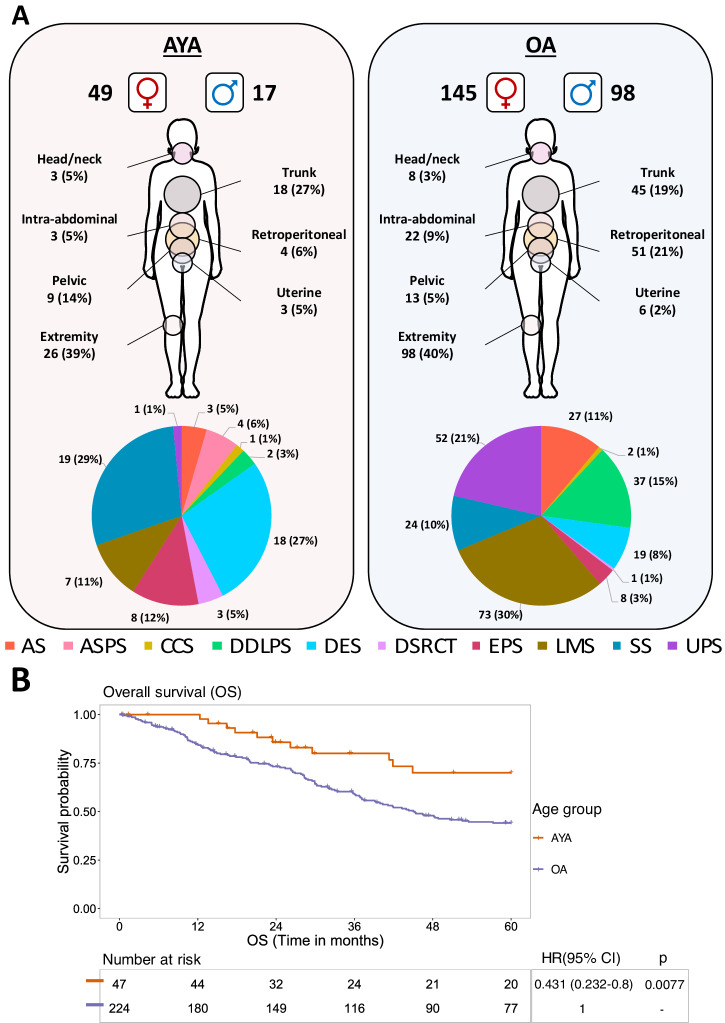
Overview of the adolescent and young adult (AYA) and older adult (OA) patients in the cohort. **a** Distribution of patient sex, anatomical site, and histological subtype within the AYA and OA cohorts. **b** Kaplan–Meier plot of overall survival (OS) for AYA and OA patients. Hazard ratio (HR), 95% confidence intervals (CI) and *p* value determined by univariable Cox regression. AS angiosarcoma, ASPS alveolar soft part sarcoma, CCS clear cell sarcoma, DDLPS dedifferentiated liposarcoma, DSRCT desmoplastic small round cell tumour, DE desmoid tumour, EPS epithelioid sarcoma, LMS leiomyosarcoma, SS synovial sarcoma, UPS undifferentiated pleomorphic sarcoma.

The cohort comprises primary tumour specimens from 309 patients (AYA = 66, OA = 243), for which comprehensive proteomic profiles by mass spectrometry (MS) have previously been generated by our laboratory^28^. Nine sarcoma subtypes are represented, including alveolar soft part sarcoma (ASPS), angiosarcoma (AS), clear cell sarcoma (CCS), dedifferentiated liposarcoma (DDLPS), desmoplastic small round cell tumour (DSRCT), epithelioid sarcoma (EPS), leiomyosarcoma (LMS), synovial sarcoma (SS) and undifferentiated pleomorphic sarcoma (UPS) (full clinicopathological information provided in Supplementary Table 1). Additionally, DES, a locally infiltrative soft tissue tumour with no metastatic potential and a relatively high incidence in the AYA age group, was included in the cohort^34^. When broken down by age groups, AYA patients are enriched for ASPS (100%) and DSRCT (75%), while OA patients are enriched for UPS (98%), DDLPS (95%), LMS (91%), AS (90%) and CCS (67%) (Fig. 1a). There are almost equal number of AYA and OA patients in SS (44% AYA), DES (49% AYA) and EPS (50% AYA). The proportion of different histological subtypes in the two age groups is reflective of real-world incidence^35^. The cohort has a female predominance (male [37%], female [63%]), with a broad distribution of different anatomical sites in each age group (Fig. 1A). Consistent with a previous study of a large cohort of ~5000 STS patients by the Scandinavian Sarcoma Group (SSG)^3^, our cohort has a higher proportion of patients with grade 3 (OA: 53%, AYA: 15%) and large tumours ≥15 cm (OA: 26%, AYA: 12%) in the OA age group (Supplementary Table 1). Similar to the SSG study, univariate Cox regression analysis showed that patients belonging to the AYA age group were at statistically significant lower risk of death compared to patients in the OA group (Hazard Ratio (HR) = 0.431, 95% Confidence Interval (CI) = 0.232-0.8, *p* = 0.0077) (Fig. 1b). There was no statistically significant difference in the two age groups for metastasis free survival (MFS) and local relapse-free survival (LRFS) (Supplementary Fig. 1). Note that DES was not included in any of the survival analysis undertaken in this study because they are not malignant.

### Analysis of the AYA and OA proteomic landscape

**Fig. 2:**
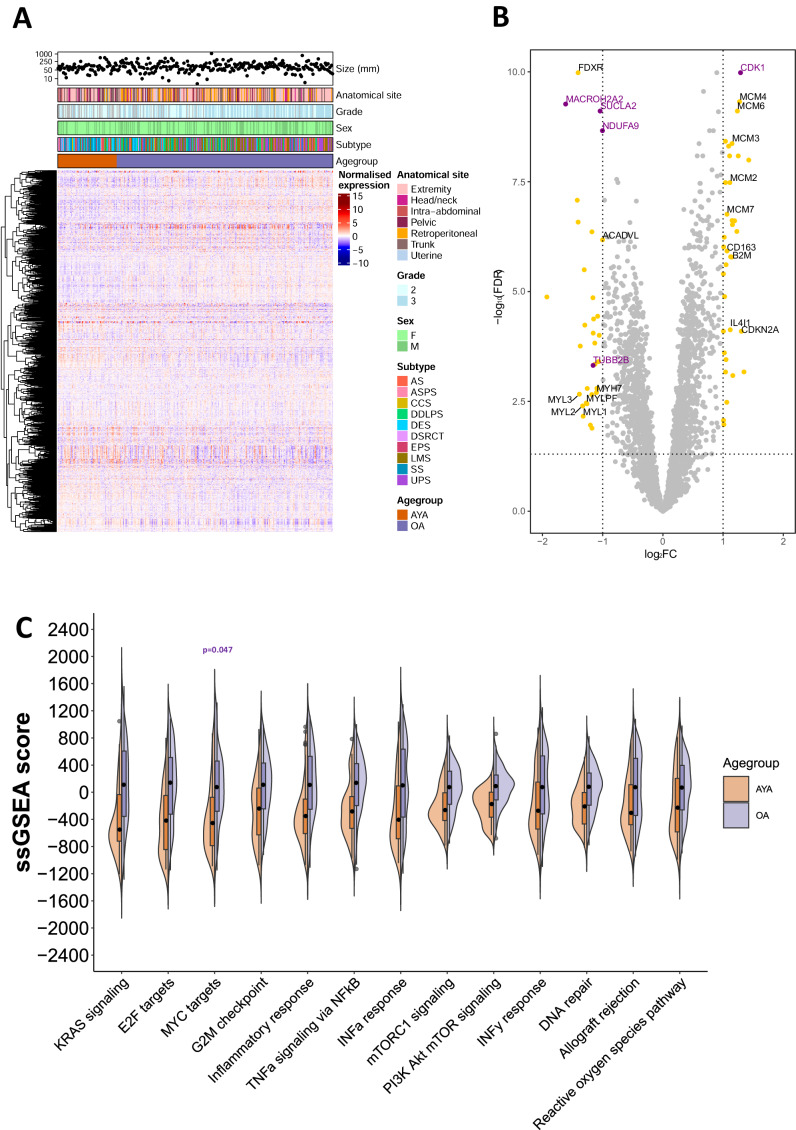
Analysis of the adolescent and young adult (AYA) and older adult (OA) proteomic landscape. **a** Annotated heatmap showing the unsupervised clustering (Pearson’s distance) of 3299 proteins across the study cohort. Patients are ordered from youngest (left) to oldest (right). From top to bottom, panels indicate tumour size, anatomical site, tumour grade, patient sex, histological subtype, and patient age group. **b** Volcano plot showing significantly upregulated proteins in AYA and OA patient tumours. Significant proteins (false discovery rate <0.05, fold change >2) determined by multiple *t*-test followed by Benjamini–Hochberg procedure are shown in yellow. Proteins that remained significant after multivariable logistic regression analysis to account for confounding factors are shown in purple. **c** Significantly enriched hallmark gene sets (*q* < 0.05) in OA patients using single sample gene set enrichment analysis (ssGSEA) scores, as determined by two-way analysis of variance (ANOVA) followed by Šidák correction. Following multivariable logistic regression to account for confounding factors, the MYC targets hallmark gene set remained significant (*p* = 0.047). Boxplots show ssGSEA scores, with boxes indicating the 25th and 75th percentile and the black dot indicating the 50th percentile. Whiskers extend from the 25th percentile − (1.5* interquartile range) to the 75th percentile + (1.5* interquartile range), and outliers are plotted as grey points. AS angiosarcoma, ASPS alveolar soft part sarcoma, CCS clear cell sarcoma, DDLPS dedifferentiated liposarcoma, DSRCT desmoplastic small round cell tumour, DES desmoid tumour, EPS epithelioid sarcoma, LMS leiomyosarcoma, SS synovial sarcoma, UPS undifferentiated pleomorphic sarcoma.

A total of 8148 proteins were identified, with 3299 proteins quantified across all samples (Fig. 2a and Supplementary Data 3). We defined the proteins that are significantly upregulated in AYA versus OA patients. Following multiple testing correction, 32 and 35 proteins were identified to be significantly upregulated in AYA or OA respectively (FDR <0.05, fold change >2) (Fig. 2b and Supplementary Data 4). Our analysis finds that OA patients harboured a significant upregulation of proteins involved in DNA replication (MCM complex), cell cycle regulation (CDK1 and CDKN2A) and immune regulation (CD163, B2M, IL4I1), while AYA patients displayed an upregulation of proteins involved in mitochondrial metabolism (NDUFA9, SUCLA2, FDXR and ACADVL) and skeletal and cardiac myosin chains (MYL1, MYL2, MYLPF and MYH7). Multivariable logistic regression analysis was performed to adjust for potential confounding factors (tumour size, grade, anatomical site, performance status and histological subtype), which led to five proteins remaining significant between the two age groups (AYA: NDUFA9, SUCLA2, TUBB2B, MACROH2A2 and OA: CDK1).

By undertaking single sample gene set enrichment analysis (ssGSEA), we show that compared to AYA patients, OA patients are significantly enriched (*q* < 0.01) for distinct biological hallmark features (Fig. 2c), including gene sets involved in cell cycle regulation (E2F targets, G2M checkpoint), oncogenic signalling (KRAS signalling, MYC targets, TNFα signalling, mTORC1 and PI3K signalling) and inflammatory pathways (inflammatory response, INFα response) (Fig. 2b). AYA patients are significantly enriched for oxidative phosphorylation (*q* = 0.004) and coagulation (*q* = 0.006) hallmarks (Supplementary Fig. 2). Multivariable logistic regression analysis was performed to adjust for potential confounding factors (tumour size, grade, anatomical site, performance status and histological subtype) which led to only the MYC targets hallmark remaining significant between the two age groups (*p* = 0.047).

### Sarcoma proteomic modules highlight distinct biological pathways in the two age groups

**Fig. 3:**
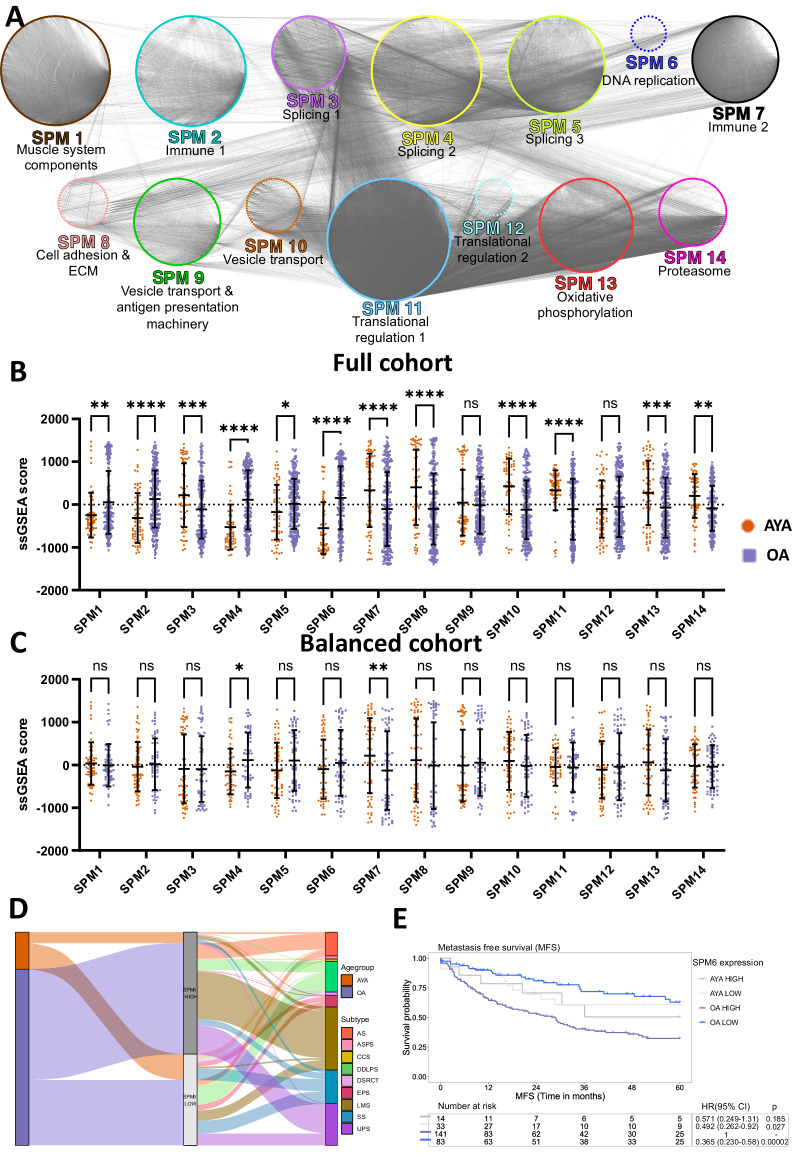
Differential expression of sarcoma proteomic modules (SPM) in adolescent and young adult (AYA) and older adult (OA) patients. **a** Protein co-expression network showing the 14 previously described SPM identified in the full proteomic cohort. Nodes indicate proteins and are coloured based on SPM membership. Edges indicate a correlation between protein expression and thickness of edges are scaled to the correlation score. **b** Plot showing single sample gene set enrichment analysis (ssGSEA) scores of the 14 SPMs for AYA and OA patients in the full cohort (*n* = 309, 66 AYA, 243 OA). **c** Plot showing single sample gene set enrichment analysis (ssGSEA) scores of the 14 SPMs for AYA and OA patients in a balanced number of cases in both age groups for each histological subtype (*n* = 120, 60 AYA, 60 OA). Error bars indicate the mean ± 1 standard deviation. Statistical significance was determined by two-way analysis of variance (ANOVA) using an uncorrected Fisher’s least significant difference (LSD) test. ****p < 0.0001, ***p < 0.001, **p < 0.01, *p < 0.05. **d** Sankey plot showing the distribution of each patient age group and histological subtype that falls into the SPM6-high and low expression groups. **e** Kaplan–Meier plot of metastasis free survival (MFS) for AYA and OA patients with high and low median expression levels of SPM6 proteins. Hazard ratio (HR), 95% confidence intervals (CI) and *p* value determined by univariable Cox regression. AS angiosarcoma, ASPS alveolar soft part sarcoma, CCS clear cell sarcoma, DDLPS dedifferentiated liposarcoma, DSRCT desmoplastic small round cell tumour, ECM extracellular matrix, EPS epithelioid sarcoma, LMS leiomyosarcoma, SS synovial sarcoma, UPS undifferentiated pleomorphic sarcoma.

We have previously identified 14 protein signatures based on sarcoma protein co-expression patterns termed Sarcoma Proteomic Modules (SPMs) which capture a broad spectrum of STS biology and transcend histological subtype (Fig. 3a and Supplementary Data 2)^28^. We first compared the enrichment of SPMs in the AYA and OA patients in the full cohort. Calculating patient-specific ssGSEA scores for each SPM showed significant differences between the two age groups for the majority of SPMs (Fig. 3b). To account for the possibility that histological subtype imbalances in the two age groups within the proteomic dataset may impact the SPM results, we repeated the ssGSEA in a sub-cohort with a balanced number of cases in both age groups for each histological subtype (*n* = 120, 60 AYA, 60 OA) (Fig. 3c). We find that SPM4 (splicing proteins) was significantly upregulated in OA patients compared to AYA patients in both the full cohort and the balanced sub-cohort. Conversely, SPM7 (immune proteins) was significantly upregulated in the AYA age group versus the OA age group in both the full cohort and the balanced sub-cohort.

SPM6 is a DNA replication module which we have previously shown to be prognostic for metastasis free survival (MFS) across the whole age range^28^. To further refine this candidate biomarker signature, we evaluated the histological subtype distribution of cases classified into SPM6-high and SPM6-low subgroups based on the median protein expression levels of the 41 proteins that make up SPM6 (Supplementary Data 2) in each of the two age groups. The Sankey plot shows that there is broad representation of histotypes in each SPM6 group, with the exception of ASPS which is only found in the SPM6-low group (Fig. 3d). In addition, there was a similar distribution of AYA or OA patients in both the SPM6-high and SPM6-low groups (Fig. 3d). Combining SPM6 and age stratified patients into four subgroups (OA-SPM6-high, OA-SPM6-low, AYA-SPM6-high, and AYA-SPM6-low) (Fig. 3e). While univariate Cox regression analysis found that the SPM6 module was able to stratify the OA patients into two groups with significantly different MFS outcomes (HR = 0.365, 95% CI 0.230–0.58, *p* = 1.97 × 10^−5^), there was no significant difference in the AYA patients (Fig. 3e). Multivariable Cox proportional hazards analysis showed that that the prognostic value of SPM6 in OA patients was independent of known prognostic factors of tumour size, grade, performance status, histological subtype and anatomical location^36,37^ (HR = 0.381, 95% CI = 0.219–0.661, *p* = 6.02 × 10^−4^) (Supplementary Table 2). This analysis demonstrates that SPM6 can be used as an independent risk stratification tool to identify a subgroup of OA patients (SPM6-high) with a high risk of distant relapse but has limited utility in AYA patients.

### Comparative functional genomic and proteomic analyses reveal prognostic significance of the spliceosome complex

**Fig. 4:**
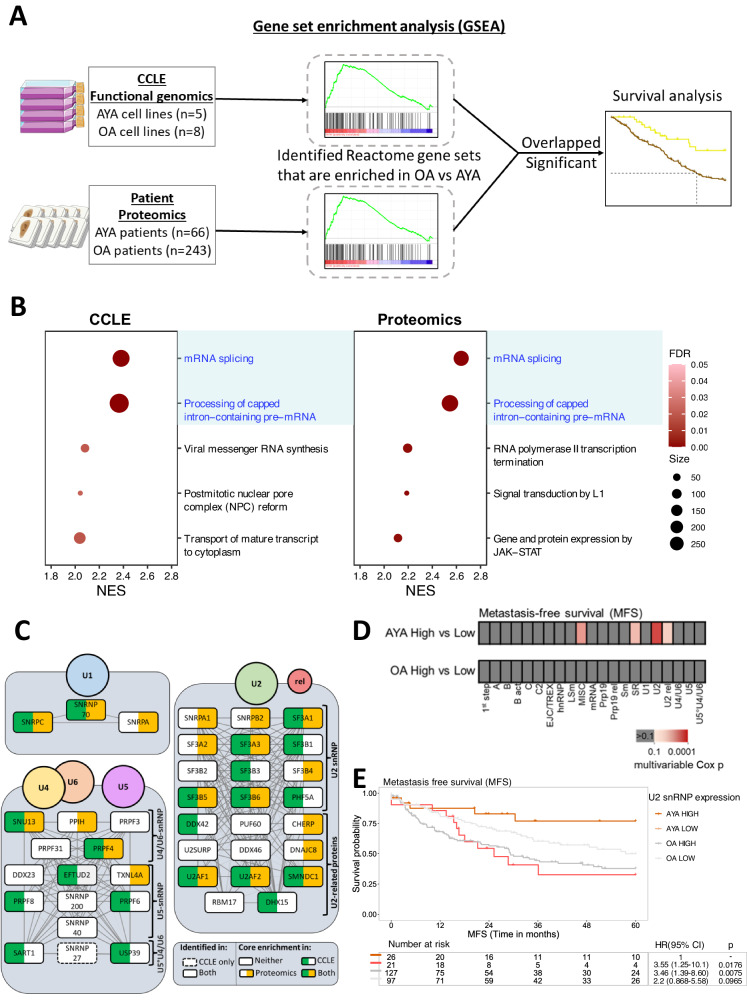
Comparative functional genomic and proteomic analysis identifies the prognostic value of the spliceosome. **a** Overview schematic of the comparative functional genomic and proteomic analysis. **b** Gene set enrichment analysis (GSEA) results showing the top five enriched reactome gene sets based on normalised enrichment score (NES) in the older adult (OA) group in the cell line functional genomics data from The Cancer Cell Line Encyclopaedia (CCLE) (left) and patient proteomics data (right). Overlapping gene sets enriched in both datasets are highlighted in blue. **c** Small nuclear ribonucleoprotein (snRNP) subunit genes that comprise the spliceosome complex found within the mRNA splicing Reactome gene set, which are identified in the CCLE cell line functional genomic and patient proteomic datasets. Genes that are only identified in the cell line functional genomic dataset are indicated with a dotted border. Genes that are core enriched as determined by GSEA leading-edge analysis in OA versus adolescent and young adult (AYA) patients in the CCLE cell line functional genomic and patient proteomic datasets are shown in green and yellow, respectively. Linkages indicate protein–protein interaction scores obtained from the STRING database, with a darker line indicating a higher score (cut-off >0.7). **d** Overview of multivariable Cox results for AYA and OA patients with high and low expression of each of the 21 subunits of the spliceosome complex as defined by Hegele et al. (based on median protein expression), and metastasis free survival (MFS). **e** Kaplan–Meier plot of MFS for AYA and OA patients with high and low expression of U2 snRNP proteins. Hazard ratio (HR), 95% confidence intervals (CI) and *p* value determined by univariable Cox regression. FDR false discovery rate.

We reasoned that biological pathways that are enriched in both CRISPR-based functional genomics and MS-based proteomics datasets may yield useful candidate drug targets and biomarkers for AYA and OA patients. Here, we sought to compare the pathway information gained from in vitro functional genomic data in a panel of sarcoma cell lines with the proteomic dataset generated from soft tissue tumour patients (Fig. 4a). We first undertook an analysis of the genome-scale CRISPR-Cas9 loss-of-function screen data focusing on the NRSTS panel of cell lines within the CCLE database^31,38^. We identified 13 NRSTS cell lines where clinical information of patient age was available (AYA:*n* = 5, OA:*n* = 8, Supplementary Data 1) and undertook gene set enrichment analysis (GSEA) using the Reactome pathway gene sets to determine the biological pathways with selective dependencies in OA compared to AYA lines (and vice versa). Separately, we performed GSEA on the proteomic dataset of 309 soft tissue tumour patients to define differential pathways that are enriched in the two age groups. Comparing the top hits that were significantly enriched in the OA group in both the CCLE functional genomics and patient-derived proteomic datasets identified multiple overlapping shared gene sets related to mRNA splicing (Fig. 4b), while gene sets comprising the respiratory electron transport were significantly enriched in both datasets in the AYA patients (Supplementary Fig. 3). To account for the possibility that histological subtype imbalances in the two age groups within the proteomic dataset may impact the GSEA results, we repeated the GSEA of the proteomic data in two sub-cohorts: (A) SS cases only (*n* = 43, 19 AYA, 24 OA) and (B) Balanced number of cases in both age groups for each histological subtype (*n* = 120, 60 AYA, 60 OA). When GSEA was performed in each of these sub-cohorts, the mRNA splicing gene sets remained in the top two hits that were enriched in the OA population compared to the AYA group (Supplementary Fig. 4), confirming that the enrichment of this biological pathway was age-specific and not dependent on histological subtype differences between the two age groups.

Given that the OA and AYA cell lines harboured distinct dependencies within the mRNA splicing gene sets, we hypothesised that components in this pathway may serve as prognostic signatures for patients in the two age groups. In particular, we focused on the spliceosome complex, which regulates the removal of introns from precursor mRNA during the splicing process (Fig. 4c). The spliceosome is a large macromolecular complex that is comprised of >200 splicing factors that vary in their composition in a spatiotemporal manner^39^. We first demonstrated that ssGSEA scores for the spliceosome complex were significantly upregulated in OA patients compared to AYA patients in the full proteomic cohort (Supplementary Fig. 5A). We then undertook the same analysis in a cohort of SS cases only or a cohort comprising a balanced number of cases in both age groups for each histological subtype. In both analyses, ssGSEA scores for the spliceosome complex remain significantly higher in OA patients compared to AYA patients (Supplementary Fig. 5B, C), demonstrating that the upregulation of this complex is age-specific.

We and others have previously shown that co-regulation of splicing factors is important in the pathology of mesenchymal and epithelial tumours^27,40,41^. As an exemplar, Fig. 4c shows the proteins in the U1, U2, U4/5/6 small nuclear ribonucleoprotein (snRNP) subunits of the spliceosome complex that are found in OA and AYA patients in the GSEA of either the CCLE functional genomics dataset or the patient proteomic dataset. We systematically assessed the prognostic value of proteins that make up each of the spliceosome functional component subunits as defined by ref. ^42^. Out of a total of 21 spliceosome subunits found in our dataset, only the U2 snRNP and a non-core miscellaneous (MISC) group of splicing factors were identified to be prognostic for MFS in AYA patients in multivariable Cox analysis (Fig. 4d). Whereas there was no significant difference in MFS between the two age groups (Supplementary Fig. 1), when categorised by median expression levels of U2 snRNP proteins (*n* = 12 proteins, Fig. 4c), AYA patients with high U2 snRNP expression (U2-high) had superior MFS outcomes compared to those with low U2 snRNP levels (U2-low) (multivariable: HR = 4.03, 95% CI = 1.13–14.4, *p* = 0.0319) (Fig. 4e and Supplementary Table 3). No spliceosome subunit protein signatures were identified to be prognostic for OA in multivariable Cox analysis. These findings highlight the utility of comparative analysis of functional genomics data with proteomic profiling as a means of defining new prognostic factors in AYA patients.

## Discussion

AYA patients with soft tissue tumours are an understudied age group with disparities in treatment and survival outcomes, where improvements in 5-year survival rates over the past two decades have lagged behind other age groups^4^. Furthermore, the use of intensive multi-modal therapy often leads to chronic health conditions and secondary malignancies in AYA patients^43,44^. Rather than the current “one size fits all” approach where AYA patients, in particular those with NRSTS, are offered treatments which have been optimised in OA, tailored strategies using targeted agents and risk stratification tools could have a substantial impact on survivorship and management of late effects. As a result of the under-representation of AYA patients in most molecular and biological studies in all cancer types, including STS^11^, there is a poor knowledge of the biological pathways that are unique to the AYA age group, which is an obstacle to developing precision medicine approaches for these patients. Notably, each of the large-scale proteomic profiling studies published thus far by The Clinical Proteomic Tumour Analysis Consortium (CPTAC) include less than 10 AYA patients^45–52^. Here, we present a large-scale analysis of the proteomic features of AYA and OA patients with soft tissue tumours. We show that there are inherent biological and pathway differences in the two age groups, which are maintained even when confounding variables such as tumour grade, size and histological subtypes are considered. We further demonstrate that comparative analysis of in vitro functional genomic data in a panel of NRSTS cell lines with the patient-level proteomic data leads to the prioritisation of age-specific vulnerabilities and independent prognostic factors, which provide new avenues for personalised treatment of AYA patients.

Several age-associated pan-cancer genomic analyses have shown that aging is associated with chronic inflammation and reprogramming of the immune cell landscape^53^. Our study finds that OA patients with soft tissue tumours are enriched in proteins involved in inflammatory response and INFα signalling hallmarks. This is consistent with a previous report by ref. ^17^, who demonstrated using GSEA and immune cell deconvolution of transcriptomic data that sarcoma patients that are <50 years of age have lower interferon responses and lymphocyte infiltration than those >50 years. We also determined that OA patients are enriched in proteins involved in cell cycle regulation, including the E2F targets and G2M checkpoint hallmarks. In agreement with our study, Chatsirisupachai et al., has shown in a pan-cancer analysis that mutations and somatic copy number alterations of genes within the cell cycle pathway are strongly enriched in tumours from older patients^16^. Interestingly, our data indicates that tumours from AYA patients harbour elevated levels of proteins involved in mitochondrial metabolism and the oxidative phosphorylation pathway, which could be indicative of metabolic rewiring in younger patients. Future investigation on the functional role of metabolic rewiring in STT subtypes in this age group is warranted^54^. Given several confounding factors contribute to biological differences between the AYA and OA age groups (for instance, tumour size, grade and histological subtype), following multivariable logistic regression to account for these factors, only the MYC targets hallmark remained significant. Our findings are in line with a recent study of age-specific proteomic features in colorectal cancer, which similarly showed enrichment of MYC targets in colorectal patients above 50 years of age^55^.

A previous analysis of genome-scale CRISPR-Cas9-based loss of function screening data in a panel of paediatric cancer cell lines identified vulnerabilities that were distinct from cell lines derived from adult patients^56^, suggesting that oncology drugs that are used in adult patients may not always be applicable to childhood cancers. In this study, we focused on the pathway vulnerabilities that are specific to sarcoma cell lines in either the AYA or OA age groups and undertook analysis to compare the GSEA outputs from the genome-scale CRISPR screening data with the patient-level proteomics data. Our analysis finds that in vitro pathway dependencies observed in sarcoma cell lines derived from patients of different age groups correspond to significantly higher expression levels of pathway proteins in either AYA (respiratory electron transport) or OA (mRNA splicing) patients. Sarcomas are a group of diseases of unmet need with a lack of effective therapies and novel agents. Investigational drugs that target components for each of these pathways are available for repurposing^57–59^ and should be evaluated in prospective studies in the different age groups. Our data further suggest that high protein expression levels of these proteins in sarcoma tissue specimens may facilitate the selection of patients who are most likely to benefit from these investigational agents and therefore should be incorporated as candidate biomarkers in clinical trial design. It should be noted that this analysis relies on the functional genomics dataset from CCLE, which suffers from several limitations. CCLE comprise immortalised cell lines which have previously been shown to include sarcoma lines which may not be fully representative of the tumours from which they were derived due to adaptation to long-term culture conditions^60,61^. Furthermore, there are multiple reports of inconsistencies and discordance between different functional genomics screening efforts using the same cell lines (for example, CCLE versus the Genomics of Drug Sensitivity in Cancer (GDSC) databases)^62–64^. It is, therefore, important to acknowledge that while functional experiments are beyond the scope of this study, there is a need for future functional validation of some of our findings in an independent set of patient-derived AYA NRSTS cell line models.

Despite optimal clinical management, a substantial proportion of NRSTS patients (up to 50%) with localised disease experience distant relapse following surgery^15^. Stratification of these high-risk patients has been limited to the use of nomograms, which consider known prognostic factors including tumour grade, size, histological subtype and age, amongst other variables^65–67^. There are currently very few molecular prognostic signatures for NRSTS and none which are optimised for AYA patients^68^. Here, we show that specific subunits of the spliceosome complex are independent prognostic factors in AYA patients. In particular, AYA patients with low tumour protein expression levels of the U2 snRNP spliceosome subunit are at higher risk of developing metastasis compared to those with high expression levels. These protein signatures have potential utility as precision medicine tools to tailor more aggressive treatment strategies such as peri-operative chemo/radiotherapy in AYA patients that are predicted to have higher risk of distant relapse. Conversely, low-risk AYA patients may be spared potential overtreatment, thereby reducing the risk of chronic health conditions and late effects. Mechanistically, it is not clear why AYA tumours with reduced spliceosome levels appear to have more aggressive features and future functional experiments are required to dissect the role of individual splicesome protein components in AYA sarcoma cell lines. Our study further highlights the importance of performing age-specific studies to delineate biomarkers tailored for the clinical management of AYA patients.

We acknowledge several limitations of this study. This is a retrospective cohort, which is prone to selection bias therefore, the study is hypothesis generating and our findings need to be validated in independent cohorts. Soft tissue tumours comprise a broad range of histological subtypes, and our study is limited to 10 and 7 histologies in the proteomic and functional genomics datasets, respectively. Future studies which include wider histological subtype representation is needed to determine if our findings are generalisable to all AYA patients, although this may be challenging given the limited number of publicly available AYA NRSTS cell line models available for functional studies^69^. There is a notable imbalance of subtypes between the two age groups in our cohort where tumours comprising complex karyotypes are prevalent in OA while those with simple genomes are enriched in the AYA group. It is important to highlight that this is reflective of the real-world incidence, with some sarcoma subtypes more likely to arise in younger patients or vice versa^35^. This is a key study limitation, and where possible, we have accounted for these differences using multivariable regression analysis to identify age-specific effects. Since our study does not include a comparative analysis of proteomic profiles of normal tissue from AYA and OA individuals, we cannot exclude the possibility that some of the enriched protein signatures and pathways identified in this study are the result of physiological aging rather than being tumour-specific. Despite this limitation, our data identify age-specific protein signatures with prognostic value in MFS in both AYA and OA patients which is indicative of pathological disease relevance.

In summary, we have undertaken a deep analysis of the biological differences in the proteomic profiles of soft tissue tumours from patients in the AYA and OA age groups. We highlight important protein-specific pathways and genetic vulnerabilities that are enriched in AYA patients and identify age-specific prognostic signatures to facilitate tailored clinical management of this underserved patient group.

### Supplementary information

Supplementary Information

Description of Additional Supplementary Files

Supplementary Data 1

Supplementary Data 2

Supplementary Data 3

Supplementary Data 4

Supplementary Data 5

Reporting Summary

## Data Availability

The raw proteomic data generated in this study have been deposited in the ProteomeXchange Consortium via the PRIDE partner repository^70,71^ with the dataset identifier PXD036226. The normalised proteomic dataset used for the study is provided as Supplementary Data 3. The clinical data is available under restricted access due to data privacy legislation, access can be obtained by contacting the corresponding author (P.H.H.) and will require researchers to sign a data access agreement with the Institute of Cancer Research after approval by the Data Access Committee (DAC) of the Institute of Cancer Research. The DAC will determine the length of permitted access, with an expected response time frame of 2 weeks for access requests. The genome-scale CRISPR-Cas9 screening data of cell lines (CRISPRGeneEffect DepMap Public 22Q4) is available from the Cancer Cell Line Encyclopaedia (CCLE) portal (https://sites.broadinstitute.org/ccle). Additional data used for figures are provided with this paper in Supplementary Data 5.
