# Supplementary material for: Proteomic features of soft tissue tumours in adolescents and young adults
Source: Commun Med (Lond). 2024 May 18;4:93. doi: 10.1038/s43856-024-00522-x (PMC11102500; doi:10.1038/s43856-024-00522-x)
Supplement: Supplementary file 1 — Supplementary Information [file 43856_2024_522_MOESM1_ESM.pdf]

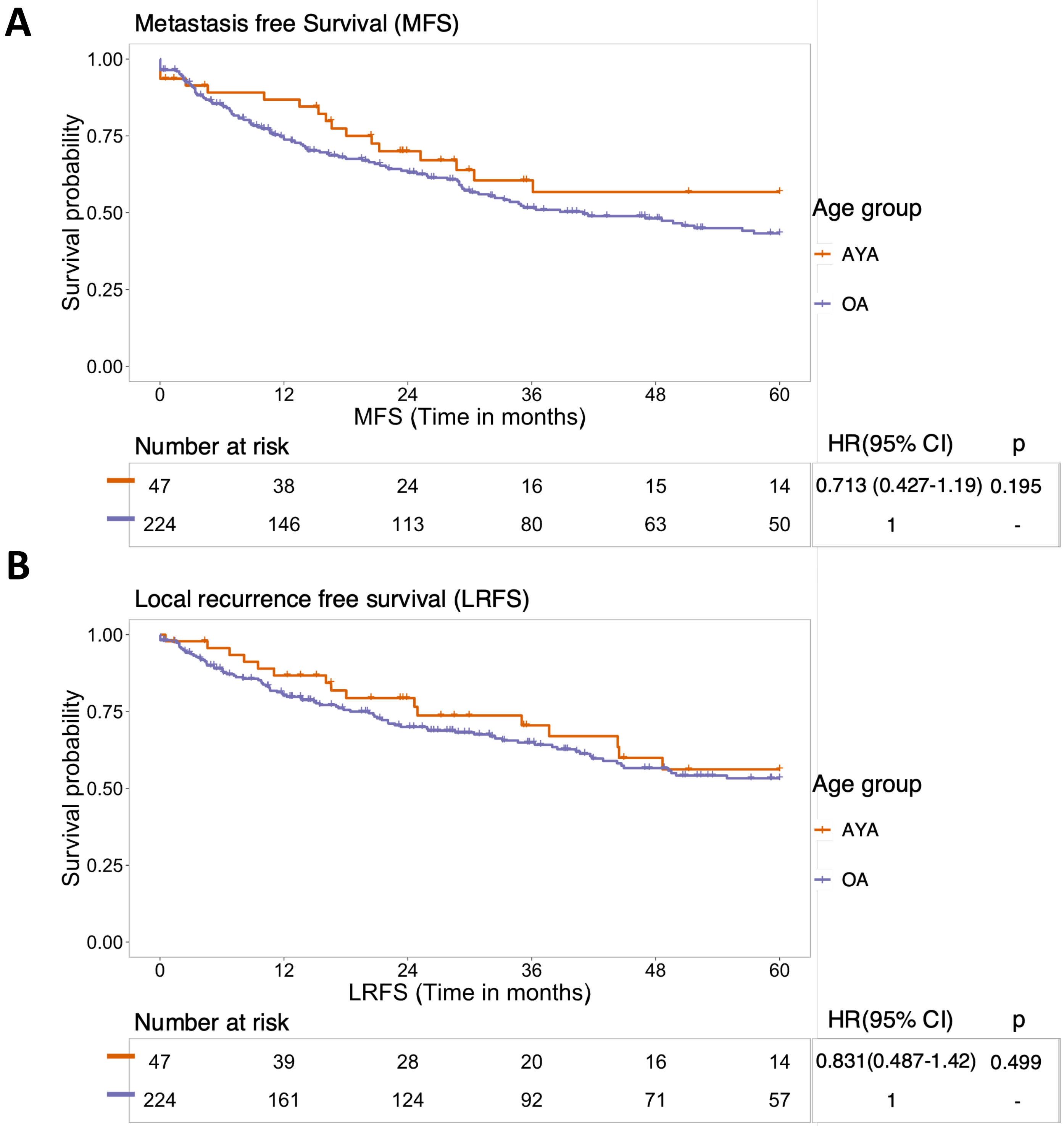

Supplementary Figure 1. Kaplan–Meier plot of (a) metastasis free survival (MFS), and (b) local recurrence free survival (LRFS) for adolescent and young adult (AYA) and older adult (OA) patients. Hazard ratio (HR), 95% confidence intervals (CI) and p-value determined by univariable Cox regression.

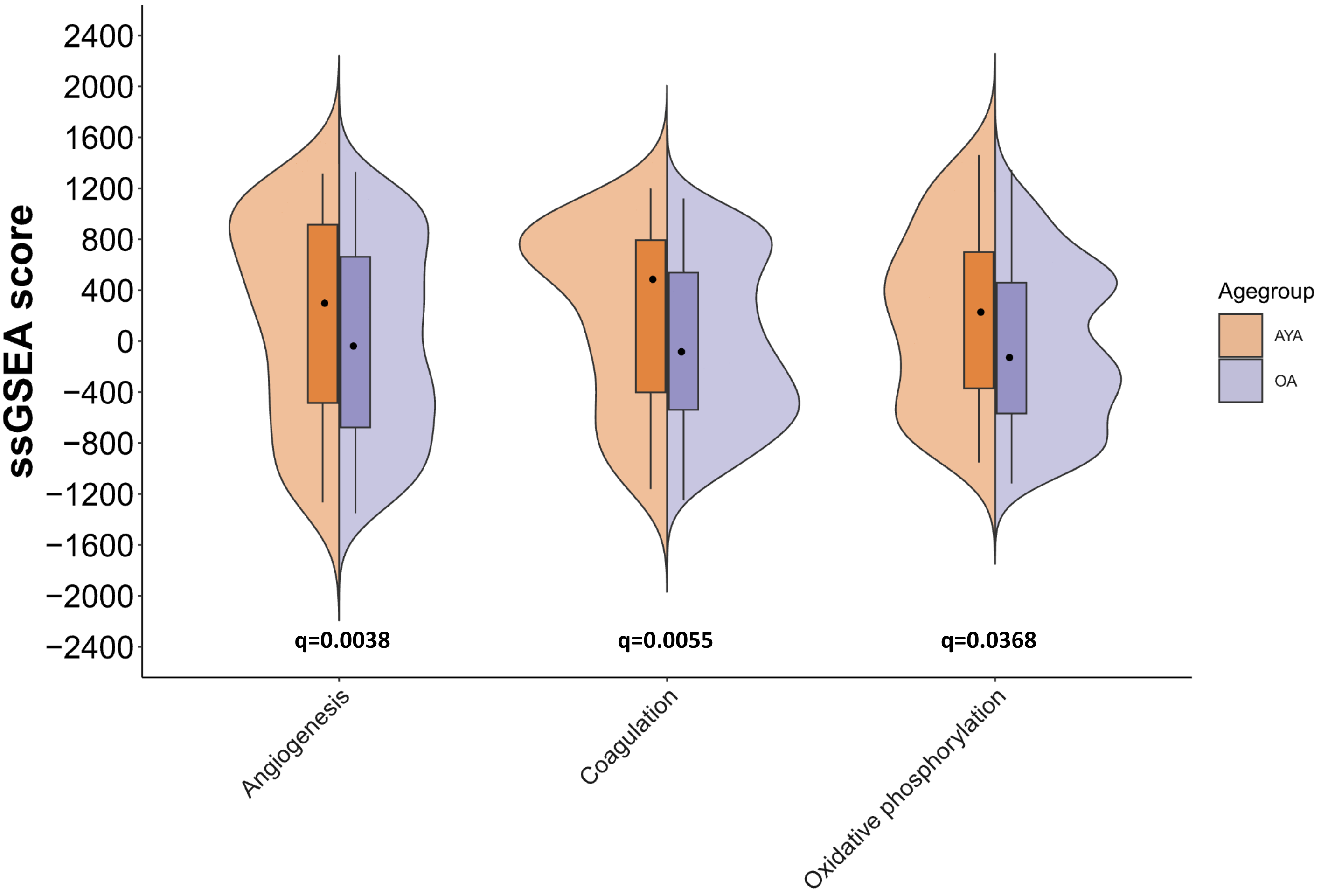

Supplementary Figure 2. Significantly enriched hallmark gene sets (q<0.05) in AYA patients using single sample gene set enrichment analysis (ssGSEA) scores, as determined by two-way analysis of variance (ANOVA) followed by Šidák correction. Boxplots shows ssGSEA scores, with boxes indicating 25th and 75th percentile and the black dot indicating the 50th percentile. Whiskers extend from 25th percentile-(1.5* interquartile range) to 75th percentile+(1.5* interquartile range), and outliers plotted as grey points.

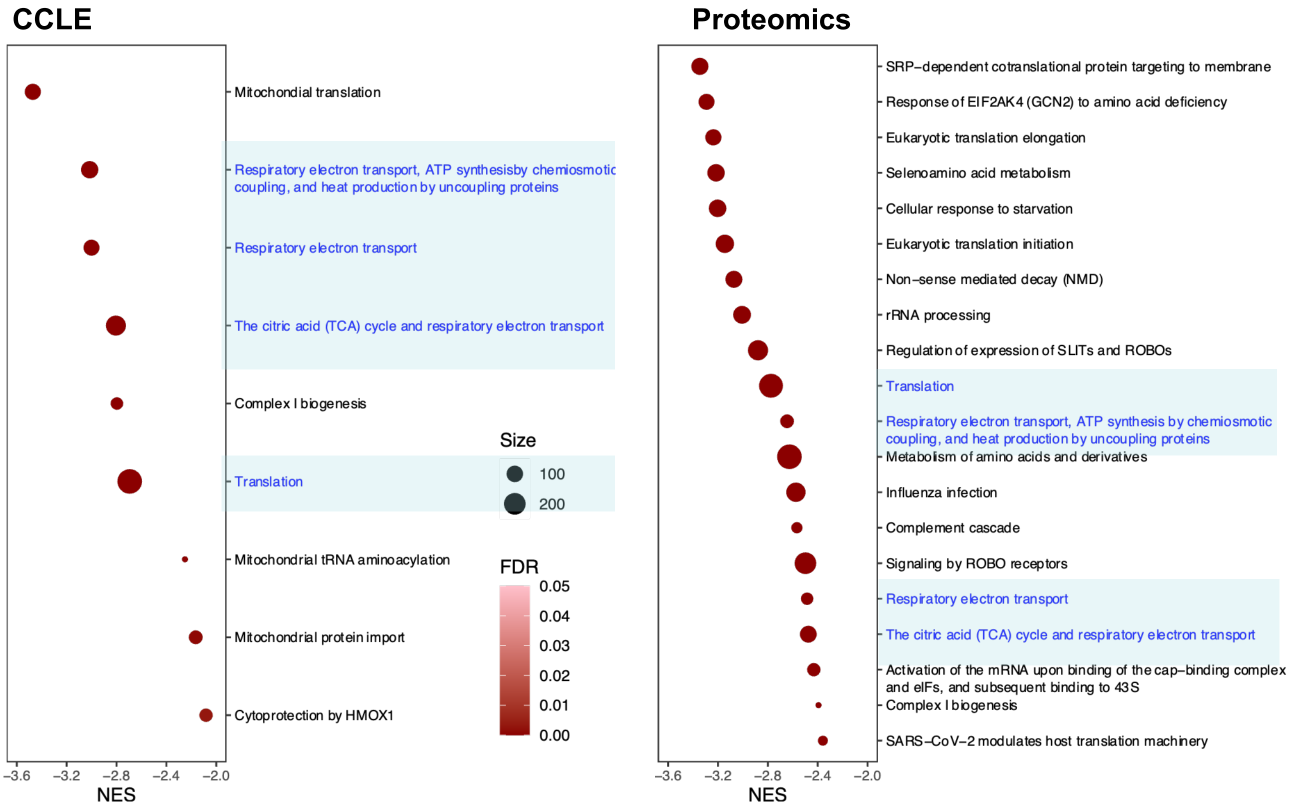

Supplementary Figure 3. Gene set enrichment analysis (GSEA) results showing enriched Reactome gene sets based on normalised enrichment score (NES) in the adolescent and young adult (AYA) group in the Cancer Cell Line Encyclopaedia (CCLE) functional genomics data (all enriched gene sets) and patient proteomics data (top 20 enriched gene sets). Overlapping gene sets enriched in both datasets are highlighted in blue.

A

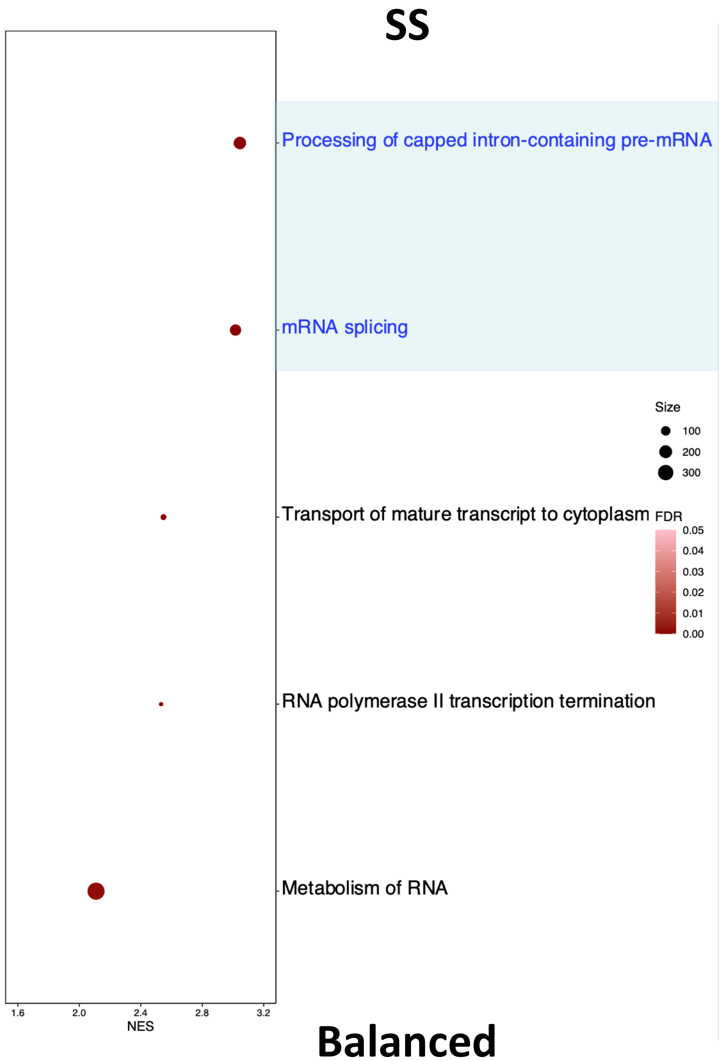
SS
Processing of capped intron-containing pre-mRNA
mRNA splicing
Transport of mature transcript to cytoplasm
RNA polymerase II transcription termination
Metabolism of RNA
Size
100
200
300
FDR
0.05
0.04
0.03
0.02
0.01
0.00
1.6
2.0
2.4
2.8
3.2
NES

B

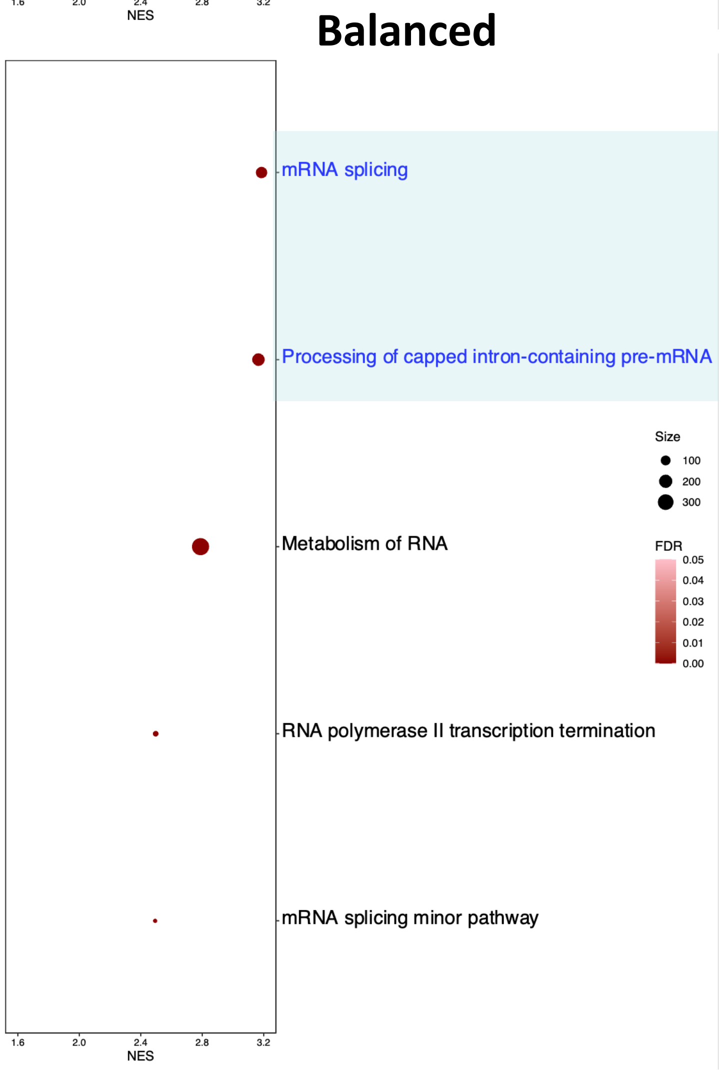
Balanced
mRNA splicing
Processing of capped intron-containing pre-mRNA
Metabolism of RNA
RNA polymerase II transcription termination
mRNA splicing minor pathway
Size
100
200
300
FDR
0.05
0.04
0.03
0.02
0.01
0.00
1.6
2.0
2.4
2.8
3.2
NES

Supplementary Figure 4. Gene set enrichment analysis (GSEA) results showing the top 5 enriched Reactome gene sets based on normalised enrichment score (NES) in the older adult (OA) group in the patient proteomics data compared to adolescent and young adults (AYA) group for (a) Synovial sarcoma (SS) cases only (n=43, 19 AYA, 24 OA) and (b) a balanced number of cases in both age groups for each histological subtype (n=120, 60 AYA, 60 OA). mRNA splicing and processing of capped intron-containing pre-mRNA gene sets (highlighted in blue) remained as the top 2 enriched hits.

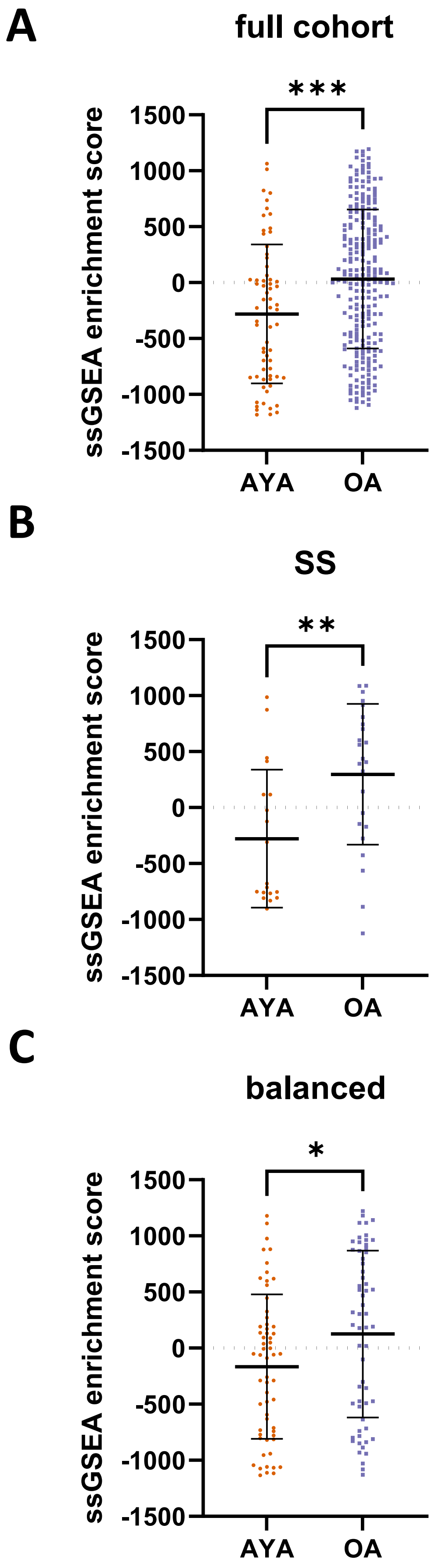

A
full cohort
***
ssGSEA enrichment score
1500
1000
500
0
-500
-1000
-1500
AYA
OA
B
SS
**
ssGSEA enrichment score
1500
1000
500
0
-500
-1000
-1500
AYA
OA
C
balanced
*
ssGSEA enrichment score
1500
1000
500
0
-500
-1000
-1500
AYA
OA

Supplementary Figure 5. Single sample gene set enrichment analysis (ssGSEA) scores for the KEGG spliceosome gene set for adolescent and young adult (AYA) and older adult (OA) patients in (a) the full cohort (n=309, 66 AYA, 243 OA), (b) synovial sarcoma (SS) cases only (n=43, 19 AYA, 24 OA) and (c) a balanced number of cases in both age groups for each histological subtype (n=120, 60 AYA, 60 OA). Error bars indicates the mean ± 1 standard deviation. Statistical significance was determined by two-tailed unpaired t-test. *** p<0.001, ** p<0.01, * p<0.05.

Supplementary Table 1. Clinicopathological features of the study cohort and associations with adolescent and young adult (AYA) and older adult (OA) age groups. AS = angiosarcoma, ASPS = alveolar soft part sarcoma, CCS = clear cell sarcoma, DDLPS = dedifferentiated liposarcoma, DSRCT = desmoplastic small round cell tumour, DES = desmoid tumour, EPS = epithelioid sarcoma, LMS = leiomyosarcoma, SS = synovial sarcoma, UPS = undifferentiated pleomorphic sarcoma.

Variable		Total	AYA	OA	Test results			
		n=309	n=66	n=243	Test performed	χ-squared	degrees of freedom	p value
Anatomical site [n(%)]	Extremity	124 (40.1)	26 (39.4)	98 (40.3)	Chi-square	15.77	6	**0.0151**
	Head/neck	11 (3.6)	3 (4.5)	8 (3.3)				
	Intra-abdominal	25 (8.1)	3 (4.5)	22 (9.1)				
	Pelvic	22 (7.1)	9 (13.6)	13 (5.3)				
	Retroperitoneal	55 (17.8)	4 (6.1)	51 (21.0)				
	Trunk	63 (20.4)	18 (27.3)	45 (18.5)				
	Uterine	9 (2.9)	3 (4.5)	6 (2.5)				
Subtype [n(%)]	AS	30 (9.7)	3 (4.5)	27 (11.1)	Chi-square	87.35	9	**<0.0001**
	ASPS	4 (1.3)	4 (6.1)	-				
	CCS	3 (1.0)	1 (1.5)	2 (0.8)				
	DDLPS	39 (12.6)	2 (3.0)	37 (15.2)				
	DES	37 (12.0)	18 (27.3)	19 (7.8)				
	DSRCT	4 (1.3)	3 (4.5)	1 (0.4)				
	EPS	16 (5.2)	8 (12.1)	8 (3.3)				
	LMS	80 (25.9)	7 (10.6)	73 (30.0)				
	SS	43 (13.9)	19 (28.8)	24 (9.9)				
	UPS	53 (17.2)	1 (1.5)	52 (21.4)				
Sex [n(%)]	Female	194 (62.8)	49 (74.2)	145 (59.7)	Chi-square	4.717	1	**0.0299**
	Male	115 (37.2)	17 (25.8)	98 (40.3)				
Tumour depth [n(%)]	Deep	250 (80.9)	61 (92.4)	189 (77.8)	Chi-square	7.441	2	**0.0242**
	Superficial	54 (17.5)	5 (7.6)	49 (20.2)				
	unknown	5 (1.6)	-	5 (2.1)				
Grade [n(%)]	2	115 (37.2)	28 (42.4)	87 (35.8)	Chi-square	45.81	2	**<0.0001**
	3	139 (45.0)	10 (15.2)	129 (53.1)				
	unknown	55 (17.8)	28 (42.4)	27 (11.1)				
Performance status [n(%)]	0	158 (51.1)	49 (74.2)	109 (44.9)	Chi-square	19.79	4	**0.0005**
	1	82 (26.5)	11 (16.7)	71 (29.2)				
	2	16 (5.2)	-	16 (6.6)				
	3	5 (1.6)	-	5 (2.1)				
	unknown	48 (15.5)	6 (9.1)	42 (17.3)				
Tumour margin [n(%)]	R0	135 (43.7)	25 (37.9)	110 (45.3)	Chi-square	1.982	3	0.5762
	R1	151 (48.9)	34 (51.5)	117 (48.1)				
	R2	4 (1.3)	1 (1.5)	3 (1.2)				
	unknown	19 (6.1)	6 (9.1)	13 (5.3)				
Log tumour size (mm) [n(%)]	<4	73 (23.6)	18 (27.3)	55 (22.6)	Chi-square	12.27	3	**0.0065**
	4-5	164 (53.1)	38 (57.6)	126 (51.9)				
	>5	70 (22.7)	8 (12.1)	62 (25.5)				
	unknown	2 (0.6)	2 (3.0)	-				

Supplementary Table 2. Multivariable cox regression with two-sided Wald test assessing metastasis free survival (MFS) of adolescent and young adult (AYA) and older adult (OA) patients categorised as high and low expression of sarcoma proteomic module 6 (SPM6). HR = hazard ratio; CI = confidence interval. AS = angiosarcoma, DDLPS = dedifferentiated liposarcoma, EPS = epithelioid sarcoma, LMS = leiomyosarcoma, SS = synovial sarcoma, UPS = undifferentiated pleomorphic sarcoma.

			**Multivariable analysis (MFS)**	
	**Variable**	**n**	**HR (95% CI)**	**p.value**
**SPM 6 group**	**OA HIGH (reference)**	141	-	-
	**OA LOW**	83	**0.381 (0.219-0.661)**	**6.02E-04**
	**AYA HIGH**	14	0.73 (0.265-2.01)	0.543
	**AYA LOW**	33	**0.324 (0.122-0.859)**	**0.0235**
**Subtype**	**LMS (reference)**	80	-	-
	**UPS**	53	1.12 (0.616-2.04)	0.708
	**SS**	43	1.18 (0.549-2.52)	0.676
	**DDLPS**	39	0.537 (0.22-1.31)	0.172
	**AS**	30	**3.11 (1.27-7.62)**	**0.0131**
	**EPS**	15	**6.6 (2.6-16.7)**	**7.04E-05**
	**Other**	11	**4.99 (1.52-16.4)**	**0.00812**
**Anatomical site**	**Extremity (reference)**	115	-	-
	**Pelvic**	20	1.22 (0.565-2.64)	0.612
	**Trunk**	41	0.656 (0.3-1.43)	0.291
	**Intra-abdominal**	21	1.66 (0.831-3.3)	0.152
	**Retroperitoneal**	55	0.81 (0.405-1.62)	0.553
	**Head/neck**	10	1.02 (0.294-3.54)	0.976
	**Uterine**	9	1.95 (0.586-6.52)	0.275
**Sex**	**F (reference)**	165	-	-
	**M**	106	1.24 (0.805-1.92)	0.328
**Log[tumour size] (mm)**	**group4-5 (reference)**	139	-	-
	**<4**	65	**0.336 (0.186-0.607)**	**2.95E-04**
	**>5**	65	1.03 (0.601-1.76)	0.916
	**missing**	2	0.913 (0.0934-8.92)	0.937
**Performance status**	**ps0 (reference)**	130	-	-
	**ps1**	78	**1.59 (1.01-2.49)**	**0.0434**
	**ps2-3**	21	1.38 (0.626-3.04)	0.426
	**missing**	42	1.62 (0.928-2.84)	0.0893
**Grade**	**grade3 (reference)**	139	-	-
	**grade2**	114	0.642 (0.408-1.01)	0.0556
	**missing**	18	0.485 (0.176-1.34)	0.163

Supplementary Table 3. Multivariable cox regression with two-sided Wald test assessing metastasis free survival (MFS) of adolescent and young adult (AYA) and older adult (OA) patients categorised as expressing high and low levels of U2 small nuclear ribonucleoprotein (snRNP). HR = hazard ratio; CI = confidence interval. AS = angiosarcoma, DDLPS = dedifferentiated liposarcoma, EPS = epithelioid sarcoma, LMS = leiomyosarcoma, SS = synovial sarcoma, UPS = undifferentiated pleomorphic sarcoma.

			Multivariable analysis (MFS)	
	**Variable**	**n**	**HR (95% CI)**	**p.value**
**U2 snRNP group**	**AYA HIGH (reference)**	26	-	-
	**AYA LOW**	21	**4.03 (1.13-14.4)**	**0.0319**
	**OA HIGH**	127	**3.29 (1.05-10.3)**	**0.0415**
	**OA LOW**	97	2.7 (0.815-8.94)	0.104
**Subtype**	**LMS (reference)**	80	-	-
	**UPS**	53	0.98 (0.54-1.78)	0.946
	**SS**	43	0.853 (0.409-1.78)	0.671
	**DDLPS**	39	**0.29 (0.126-0.668)**	**0.00365**
	**AS**	30	**2.78 (1.17-6.6)**	**0.021**
	**EPS**	15	**3.13 (1.3-7.56)**	**0.0111**
	**Other**	11	2.14 (0.699-6.53)	0.183
**Anatomical site**	**Extremity (reference)**	115	-	-
	**Pelvic**	20	1.06 (0.487-2.33)	0.876
	**Trunk**	41	0.748 (0.35-1.6)	0.455
	**Intra-abdominal**	21	**1.99 (1.01-3.9)**	**0.046**
	**Retroperitoneal**	55	1.12 (0.547-2.3)	0.754
	**Head/neck**	10	1.02 (0.301-3.43)	0.979
	**Uterine**	9	2.44 (0.737-8.1)	0.144
**Sex**	**F (reference)**	165	-	-
	**M**	106	1.27 (0.827-1.96)	0.273
**Log[tumour size] (mm)**	**group4-5 (reference)**	139	-	-
	**<4**	65	**0.358 (0.197-0.65)**	**7.36E-04**
	**>5**	65	1.1 (0.641-1.89)	0.727
	**missing**	2	1.92 (0.203-18.2)	0.569
**Performance status**	**ps0 (reference)**	130	-	-
	**ps1**	78	1.42 (0.908-2.23)	0.124
	**ps2-3**	21	1.05 (0.469-2.35)	0.906
	**missing**	42	1.4 (0.812-2.42)	0.225
**Grade**	**grade3 (reference)**	139	-	-
	**grade2**	114	**0.513 (0.33-0.797)**	**0.00301**
	**missing**	18	**0.325 (0.118-0.9)**	**0.0305**
