# Supplementary material for: Proteomic features of soft tissue tumours in adolescents and young adults
Source: Commun Med (Lond). 2024 May 18;4:93. doi: 10.1038/s43856-024-00522-x (PMC11102500; doi:10.1038/s43856-024-00522-x)
Supplement: Supplementary file 2 — Description of Additional Supplementary Files [file 43856_2024_522_MOESM2_ESM.pdf]

Description of Additional Supplementary Files

File type: Supplementary Data 1.

File description- List of nonrhabdomyosarcoma soft tissue sarcoma (NRSTS) cell lines included in the study. AYA = adolescent and young adult, OA = older adult.

File type: Supplementary Data 2.

File description- Sarcoma proteome module (SPM) membership.

File type: Supplementary Data 3.

File description- Normalised proteomic profiling data for (a) adolescent and young adult (AYA) and (b) older adult (OA) patients.

File type: Supplementary Data 4.

File description- Significantly differentially expressed proteins in (a) adolescent and young adult (AYA) and (b) older adult (OA) patients. Fold change of protein expression between OA compared to AYA. Significance was calculated by multiple t-test and adjusted for multiple comparisons using the Benjamini-Hochberg procedure. FDR = false discovery rate.
