# Supplementary material for: Proteomic features of soft tissue tumours in adolescents and young adults
Source: Commun Med (Lond). 2024 May 18;4:93. doi: 10.1038/s43856-024-00522-x (PMC11102500; doi:10.1038/s43856-024-00522-x)
Supplement: Supplementary file 8 — Reporting Summary [file 43856_2024_522_MOESM8_ESM.pdf]

nature portfolio

Corresponding author(s): Paul Huang

Last updated by author(s): Mar 9, 2024

Reporting Summary

Nature Portfolio wishes to improve the reproducibility of the work that we publish. This form provides structure for consistency and transparency in reporting. For further information on Nature Portfolio policies, see our Editorial Policies and the Editorial Policy Checklist.

Statistics

For all statistical analyses, confirm that the following items are present in the figure legend, table legend, main text, or Methods section.

n/a	Confirmed	
☐	☒	The exact sample size (*n*) for each experimental group/condition, given as a discrete number and unit of measurement
☒	☐	A statement on whether measurements were taken from distinct samples or whether the same sample was measured repeatedly
☐	☒	The statistical test(s) used AND whether they are one- or two-sided<br>*Only common tests should be described solely by name; describe more complex techniques in the Methods section.*
☐	☒	A description of all covariates tested
☐	☒	A description of any assumptions or corrections, such as tests of normality and adjustment for multiple comparisons
☐	☒	A full description of the statistical parameters including central tendency (e.g. means) or other basic estimates (e.g. regression coefficient) AND variation (e.g. standard deviation) or associated estimates of uncertainty (e.g. confidence intervals)
☐	☒	For null hypothesis testing, the test statistic (e.g. *F*, *t*, *r*) with confidence intervals, effect sizes, degrees of freedom and *P* value noted<br>*Give P values as exact values whenever suitable.*
☒	☐	For Bayesian analysis, information on the choice of priors and Markov chain Monte Carlo settings
☒	☐	For hierarchical and complex designs, identification of the appropriate level for tests and full reporting of outcomes
☒	☐	Estimates of effect sizes (e.g. Cohen's *d*, Pearson's *r*), indicating how they were calculated

Our web collection on statistics for biologists contains articles on many of the points above.

Software and code

Policy information about availability of computer code

Data collection	Proteomic data (PXD036226) for this study was downloaded from ProteomeXchange (https://www.ebi.ac.uk/pride/archive/projects/PXD036226).<br>Genome-scale CRISPR-Cas9 screening data of cell lines (CRISPRGeneEffect DepMap Public 22Q4)was downloaded from the Cancer Cell Line Encyclopaedia (CCLE) portal (https://sites.broadinstitute.org/ccle).
Data analysis	Protein-protein interaction network analysis was performed using Cytoscape (v.3.9.1) and the STRING database (v11.0).<br>Gene set enrichment analysis was performed with the GSEA (v20.4.0) module on the GenePattern public server.<br>Single sample gene set enrichment analysis was performed with the ssGSEA (v10.1.0) module on the GenePattern public server.<br>The remaining downstream analysis was performed in R (v.3.5.1 or later).

For manuscripts utilizing custom algorithms or software that are central to the research but not yet described in published literature, software must be made available to editors and reviewers. We strongly encourage code deposition in a community repository (e.g. GitHub). See the Nature Portfolio guidelines for submitting code & software for further information.

Data

Policy information about availability of data

All manuscripts must include a data availability statement. This statement should provide the following information, where applicable:

- Accession codes, unique identifiers, or web links for publicly available datasets
- A description of any restrictions on data availability
- For clinical datasets or third party data, please ensure that the statement adheres to our policy

The raw proteomic data generated in this study have been deposited in the ProteomeXchange Consortium via the PRIDE partner repository70,71 with the dataset identifier PXD036226 (https://www.ebi.ac.uk/pride/archive/projects/PXD036226). The clinical data is available under restricted access due to data privacy legislation, access can be obtained by contacting the corresponding author (P.H.H.) and will require researchers to sign a data access agreement with the Institute of Cancer Research after approval by the Data Access Committee (DAC) of the Institute of Cancer Research. The DAC will determine the length of permitted access, with an expected response time frame of 2 weeks for access requests. The genome-scale CRISPR-Cas9 screening data of cell lines (CRISPRGeneEffect DepMap Public 22Q4) is available from the Cancer Cell Line Encyclopaedia (CCLE) portal (https://sites.broadinstitute.org/ccle).

Human research participants

Policy information about studies involving human research participants and Sex and Gender in Research.

Reporting on sex and gender	Data on patient sex (assigned) was collected as part of this study and was used as a covariate in the analyses throughout this manuscript. Out of 309 patients, 194 were assigned female at birth and 115 male at birth. Information on gender was not collected.
Population characteristics	Proteomic data of patients with a histopathologically confirmed diagnosis of soft tissue sarcoma or desmoid tumour, and above 16 years of age at time of diagnosis, were included in the study. Soft tissue sarcoma diagnoses included angiosarcoma, alveolar soft part sarcoma, clear cell sarcoma, dedifferentiated liposarcoma, desmoplastic small round cell tumour, epithelioid sarcoma, synovial sarcoma, leiomyosarcoma, and undifferentiated pleomorphic sarcoma. Full population characteristics can be found in Table S1.
Recruitment	Recruitment is not applicable as this is a retrospective study.
Ethics oversight	This study was approved as part of the Royal Marsden Hospital (RMH) PROgnoStic and PrEdiCTive ImmUnoprofiling of Sarcomas (PROSPECTUS) study (NHS Research Ethics Committee Reference 16/EE/0213), and National Taiwan University Hospital (Research Ethics committee Reference 201912226RINB). Written informed consent was obtained from participants.

Note that full information on the approval of the study protocol must also be provided in the manuscript.

Field-specific reporting

Please select the one below that is the best fit for your research. If you are not sure, read the appropriate sections before making your selection.

☒ Life sciences ☐ Behavioural & social sciences ☐ Ecological, evolutionary & environmental sciences

For a reference copy of the document with all sections, see nature.com/documents/nr-reporting-summary-flat.pdf

Life sciences study design

All studies must disclose on these points even when the disclosure is negative.

Sample size	Proteomic data for 309 patients were included based on sample and data availability. Sample size is sufficient due to the rarity of the disease.
Data exclusions	Desmoid tumour patients were excluded from survival analyses as they are locally aggressive tumours that show no metastatic potential. Therefore overall survival, metastasis free survival and local recurrence free survival are not clinically meaningful measures of disease outcome and are not comparable with the rest of the cohort.
Replication	Proteomic measurements were taken from individual samples within the cohort, no technical replicates were performed.
Randomization	Randomization is not relevant in this retrospective study.
Blinding	Blinding is not relevant, as samples were not allocated to experimental groups.

Reporting for specific materials, systems and methods

We require information from authors about some types of materials, experimental systems and methods used in many studies. Here, indicate whether each material, system or method listed is relevant to your study. If you are not sure if a list item applies to your research, read the appropriate section before selecting a response.

Materials & experimental systems

n/a	Involved in the study
☒	☐ Antibodies
☒	☐ Eukaryotic cell lines
☒	☐ Palaeontology and archaeology
☒	☐ Animals and other organisms
☒	☐ Clinical data
☒	☐ Dual use research of concern

Methods

n/a	Involved in the study
☒	☐ ChIP-seq
☒	☐ Flow cytometry
☒	☐ MRI-based neuroimaging
